# Synacinn™: Bacterial reverse mutation test data in five histidine-requiring strains of *Salmonella Typhimurium*

**DOI:** 10.1016/j.dib.2021.107075

**Published:** 2021-05-07

**Authors:** Siti Nurazwa Zainol, Anis Fadhlina, Sri Vijaya Rentala, Manjula Yalaka, Leela Krishna Vatsavai, Renuka Pillai, Hassan Fahmi Ismail, Fadzilah Adibah Abdul Majid

**Affiliations:** a Proliv Life Sciences Sdn Bhd, D-1-15, Residensi Bistaria, Jln Ulu Kelang, Taman Ukay Bistari, 68000 Ampang, Selangor Darul Ehsan, Malaysia; b Institute of Marine Biotechnology, Universiti Malaysia Terengganu, 21030 Kuala Nerus, Terengganu Darul Iman, Malaysia; c Faculty of Pharmacy, International Islamic University Malaysia, Bandar Indera Mahkota, 25200 Kuantan, Pahang, Malaysia; d Aurigene Pharmaceutical Services Limited, Bollaram Road, Miyapur, Hyderabad-500 049, Telangana, India

**Keywords:** Botanical medicine, Synacinn^TM^, Mutagenicity, *Salmonella typhimurium*, Ames test

## Abstract

The present data described the analysis of mutagenicity in Synacinn^TM^ by assessing the point mutations occurring due to Synacinn™ exposure to five tester strains of *Salmonella typhimurium* (TA1537, TA1535, TA98, TA100 and TA102), in the presence or absence of an exogenous mammalian metabolic activation system (S9). It was conducted in two Phases - Phase I (Dose Range Finding experiment-DRF) and Phase II (Mutagenicity Assay 1 and 2). DRF and Mutagenicity Assay 1 was conducted employing plate incorporation method, while Mutagenicity Assay 2 was performed using pre-incubation method. Formulation analysis pertaining to Synacinn^TM^ was performed for both Mutagenicity Assay 1 and 2. Dose formulations were prepared fresh on each day of the experiment. Adventol 50% v/v in purified water was selected as a suitable vehicle based on the preliminary solubility test. Based on the Phase I analysis, 5 mg/plate was selected as the highest concentration of Synacinn^TM^ followed by lower concentrations of 2.5, 1.25, 0.625 and 0.313 mg/plate for the Mutagenicity Assays. Genetic integrity of all the tester strains used was confirmed by performing genotyping before their use. All the data acceptability criteria were fulfilled confirming the validity of the test.

## Specifications Table

**Table:** 

Subject	Biological sciences
Specific subject area	Microbiology: Applied Microbiology
Type of data	Table
How data were acquired	Revertant colonies grown on Minimal Agar plates were counted manually. Background bacterial lawn was observed using microscope and scored.
Data format	Raw Analyzed
Parameters for data collection	After incubation for 48-72 hours (DRF and Mutagenicity Assay 1 and 2), plates were removed from the incubator and each treatment plate was assessed for precipitation and cytotoxicity.
Description of data collection	Precipitation was graded based on the amount of visible precipitate on the plate. Cytotoxicity was assessed in terms of diminution of background bacterial lawn, presence of micro colonies and/or reduction in the number of revertant colonies. The fold increase in revertant colony counts for each test item treatment versus the vehicle control group was determined to assess the mutagenic potential of Synacinn^TM^.
Data source location	Aurigene Pharmaceutical Services Limited Bollaram Road, Miyapur Hyderabad - 500 049, Telangana, India.
Data accessibility	With the article

## Value of the Data

• These data provide information on the mutagenicity potential of SynacinnTM using five tester strains of Salmonella typhimurium (TA1537, TA1535, TA98, TA100 and TA102) in the absence and presence of an exogenous metabolic activation system (S9).
• These data are useful for researchers who want to determine the mutagenicity potential of their polyherbal formulations.
• SynacinnTM is currently approved by National Pharmaceutical Regulatory Agency (NPRA), Malaysia as traditional medicine with a general health claim. Data on the mutagenicity potential of SynacinnTM are valuable to establish the safety pharmacology for SynacinnTM and can be used in future for the development process and approval of SynacinnTM as a prescription botanical medicine for diabetes.

## 1 Data Description

All markers identified in Synacinn™ formulations (Mutagenicity assay 1 and 2: raw data as in Supplementary Table 1S) was presented in Table 1 for all dose strengths (6.25 mg/mL, 12.5 mg/mL, 25 mg/mL and 50 mg/mL). Table 2 shows the peak area of catechin from HPLC analysis of top, middle and bottom layers of Synacinn™ formulations in triplicate. In the Dose Range Finding experiment, Synacinn™ was tested for cytotoxicity and precipitation in the tester strain TA100 at concentrations ranging from 0.039 to 5 mg/plate (Table 3). No signs of precipitation or cytotoxicity (in terms of reduction of revertant colony count and or diminution of bacterial background lawn) was observed up to the highest tested concentration of 5 mg/plate. Hence, 5 mg/plate was selected as the highest dose for Phase II, followed by 4 lower concentrations separated by factor of 2. Data on the revertant colony counts and observation on the precipitation and background lawn of five selected strains obtained in Phase I and Phase II (Mutagenicity Assay 1 & 2) with or without the presence of an exogenous metabolic activation system (S9) are presented in Table 4, Table 5, Table 6, Table 7, Table 8, Table 9, Table 10, Table 11, Table 12, Table 13. Each of the *S. typhimurium* strains (TA1537, TA1535, TA98, TA100 and TA102) data is individually presented in the Tables 4–13.

**Table 1:** Identification (Retention Time) of markers in Synacinn™ formulation.

	Retention time (Mutagenicity Assay 1)				
Formulation dose	Gallic acid	Catechin	Rosmarinic acid	Andrographolide	Curcumin
6.25-25mg/mL	5.212-5.328	11.668-11.910	19.862-19.915	21.533-21.651	26.732-26.958

	Retention Time (Mutagenicity Assay 2)				
Formulation dose	Gallic Acid	Catechin	Rosmarinic Acid	Andrographolide	Curcumin

6.25-25mg/mL	5.152-5.373	11.549-11.973	19.549-19.845	21.365-21.450	26.251-26.622

**Table 2:** Peak area of catechin in Synacinn™ formulation analysis.

	Area (Mutagenicity Assay 1)			
Formulation layers	6.25 mg/mL	12.5 mg/mL	25 mg/mL	50 mg/mL
**Top-1**	50992	53350	42421	39714
**Top-2**	56718	46548	43463	39219
**Top-3**	47900	55111	40496	40956
**Middle-1**	43940	64415	42195	40151
**Middle-2**	58521	48852	41151	39475
**Middle-3**	52010	57305	41949	38905
**Bottom-1**	55076	56279	41881	40662
**Bottom-2**	51249	49904	43131	39586
**Bottom-3**	48223	53091	42098	30996
**Mean Area**	51625.4	53872.8	42087.2	38851.6
**SD**	4617.9217	5311.9905	906.6083	3018.9915
**%RSD**	8.9	9.9	2.2	7.8

	Area (Mutagenicity Assay 2)			
Formulation layers	6.25 mg/mL	12.5 mg/mL	25 mg/mL	50 mg/mL

**Top-1**	45426	53383	35934	36531
**Top-2**	45522	51243	34962	37525
**Top-3**	45784	53550	37015	35712
**Middle-1**	46193	50536	37896	36889
**Middle-2**	46473	49688	39045	37029
**Middle-3**	46819	51242	37510	36664
**Bottom-1**	46586	50209	37135	37147
**Bottom-2**	44474	52464	38408	35607
**Bottom-3**	45015	50287	36925	36165
**Mean Area**	45810.2	51400.2	37203.3	36585.4
**SD**	778.9826	1417.3561	1233.3481	651.2646
**%RSD**	1.7	2.8	3.3	1.8

**Table 3:** Data of dose range finding experiment by plate incorporation method.

DRF - Plate Incorporation					Organism:	TA100		
		No. of Revertants						
Test Item	Treatment	R1	R2	R3	Mean	SD	Observation	Fold increase
Untreated control	**-**	198	203	184	**195**	**9.8**	NR, NP	-

**Without S9**								

Adventol (50% v/v)	0	178	206	211	**198**	**17.8**	NR, NP	-
Synacinn™ (mg/plate)	0.039	218	246	237	**234**	**14.3**	NR, NP	1.18
	0.078	205	252	209	**222**	**26.1**	NR, NP	1.12
	0.156	221	243	238	**234**	**11.5**	NR, NP	1.18
	0.313	247	257	225	**243**	**16.4**	NR, NP	1.23
	0.625	251	205	209	**222**	**25.5**	NR, NP	1.12
	1.25	240	219	233	**231**	**10.7**	NR, NP	1.17
	2.5	219	232	213	**221**	**9.7**	NR, NP	1.12
	5	199	226	234	**220**	**18.3**	NR, NP	1.11
Sodium azide (µg/plate)	5	1868	1668	1750	**1762**	**100.5**	NR, NP	9.04

**With S9**								

Adventol (50% v/v)	0	187	234	191	**204**	**26.1**	NR, NP	-
Synacinn™ (mg/plate)	0.039	248	202	201	**217**	**26.9**	NR, NP	1.06
	0.078	261	220	230	**237**	**21.4**	NR, NP	1.16
	0.156	238	230	212	**227**	**13.3**	NR, NP	1.11
	0.313	201	198	200	**200**	**1.5**	NR, NP	0.98
	0.625	227	273	207	**236**	**33.8**	NR, NP	1.16
	1.25	195	236	228	**220**	**21.7**	NR, NP	1.08
	2.5	238	237	221	**232**	**9.5**	NR, NP	1.14
	5	224	231	208	**221**	**11.8**	NR, NP	1.08
2AA (µg/plate)	5	1550	1350	1456	**1452**	**100.1**	NR, NP	7.45

2AA: 2-Aminoanthracene; NR: No reduction in bacterial background lawn; NP: No precipitation

**Table 4:** Mutagenicity assay 1 by plate incorporation method on TA1537.

Mutagenicity assay 1 - Plate Incorporation					Organism:	TA1537		
		No. of Revertants						
Test Item	Treatment	R1	R2	R3	Mean	SD	Observation	Fold increase
Untreated control	**-**	12	18	10	**13**	**4.2**	NR, NP	-

**Without S9**								

Adventol (50% v/v)	0	11	11	14	**12**	**1.7**	NR, NP	-
Synacinn™ (mg/plate)	0.313	11	8	8	**9**	**1.7**	NR, NP	**0.75**
	0.625	6	10	5	**7**	**2.6**	NR, NP	**0.58**
	1.25	13	6	14	**11**	**4.4**	NR, NP	**0.92**
	2.5	10	8	13	**10**	**2.5**	NR, NP	**0.83**
	5	18	8	15	**14**	**5.1**	NR, NP	**1.17**
ICR-191 (µg/plate)	1	182	165	173	**173**	**8.5**	NR, NP	**13.31**

**With S9**								

Adventol (50% v/v)	0	12	13	17	**14**	**2.6**	NR, NP	-
Synacinn™ (mg/plate)	0.313	16	11	11	**13**	**2.9**	NR, NP	**0.93**
	0.625	14	14	10	**13**	**2.3**	NR, NP	**0.93**
	1.25	12	13	12	**12**	**0.6**	NR, NP	**0.86**
	2.5	16	8	10	**11**	**4.2**	NR, NP	**0.79**
	5	10	10	11	**10**	**0.6**	NR, NP	**0.71**
2AA (µg/plate)	5	252	216	208	**225**	**23.4**	NR, NP	**17.31**

2AA: 2-Aminoanthracene; NR: No reduction in bacterial background lawn; NP: No precipitation

**Table 5:** Mutagenicity assay 1 by plate incorporation method on TA1535.

Mutagenicity assay 1 - Plate Incorporation					Organism:	TA1535		
		No. of Revertants						
Test Item	Treatment	R1	R2	R3	Mean	SD	Observation	Fold increase
Untreated control	**-**	23	13	17	**18**	**5.0**	NR, NP	-

**Without S9**								

Adventol (50% v/v)	0	19	14	20	**18**	**3.2**	NR, NP	-
Synacinn™ (mg/plate)	0.313	17	26	15	**19**	**5.9**	NR, NP	**1.06**
	0.625	27	25	12	**21**	**8.1**	NR, NP	**1.17**
	1.25	28	26	11	**22**	**9.3**	NR, NP	**1.22**
	2.5	22	14	17	**18**	**4.0**	NR, NP	**1.00**
	5	26	18	24	**23**	**4.2**	NR, NP	**1.28**
Sodium azide (µg/plate)	5	960	1120	1026	**1035**	**80.4**	NR, NP	**57.50**

**With S9**								

Adventol (50% v/v)	0	17	9	15	**14**	**4.2**	NR, NP	-
Synacinn™ (mg/plate)	0.313	10	8	19	**12**	**5.9**	NR, NP	**0.86**
	0.625	11	10	11	**11**	**0.6**	NR, NP	**0.79**
	1.25	6	14	12	**11**	**4.2**	NR, NP	**0.79**
	2.5	12	5	9	**9**	**3.5**	NR, NP	**0.64**
	5	10	10	8	**9**	**1.2**	NR, NP	**0.64**
2AA (µg/plate)	5	169	258	196	**208**	**45.6**	NR, NP	**11.56**

2AA: 2-Aminoanthracene; NR: No reduction in bacterial background lawn; NP: No precipitation

**Table 6:** Mutagenicity assay 1 by plate incorporation method on TA98.

Mutagenicity assay 1 - Plate Incorporation					Organism:	TA98		
		No. of Revertants						
Test Item	Treatment	R1	R2	R3	Mean	SD	Observation	Fold increase
Untreated control	**-**	46	63	52	**54**	**8.6**	NR, NP	-

**Without S9**								

Adventol (50% v/v)	0	68	49	57	**58**	**9.5**	NR, NP	-
Synacinn™ (mg/plate)	0.313	69	81	58	**69**	**11.5**	NR, NP	**1.19**
	0.625	82	62	72	**72**	**10.0**	NR, NP	**1.24**
	1.25	81	64	50	**65**	**15.5**	NR, NP	**1.12**
	2.5	79	43	61	**61**	**18.0**	NR, NP	**1.05**
	5	76	57	51	**61**	**13.1**	NR, NP	**1.05**
2-Nitrofluorene (µg/plate)	5	896	912	850	**886**	**32.2**	NR, NP	**16.41**

**With S9**								

Adventol (50% v/v)	0	49	52	50	**50**	**1.5**	NR, NP	-
Synacinn™ (mg/plate)	0.313	59	52	51	**54**	**4.4**	NR, NP	**1.08**
	0.625	56	62	42	**53**	**10.3**	NR, NP	**1.06**
	1.25	65	46	58	**56**	**9.6**	NR, NP	**1.12**
	2.5	63	48	45	**52**	**9.6**	NR, NP	**1.04**
	5	63	58	53	**58**	**5.0**	NR, NP	**1.16**
2AA (µg/plate)	5	1462	1650	1572	**1561**	**94.5**	NR, NP	**28.91**

2AA: 2-Aminoanthracene; NR: No reduction in bacterial background lawn; NP: No precipitation

**Table 7:** Mutagenicity assay 1 by plate incorporation method on TA100.

Mutagenicity assay 1 - Plate Incorporation					Organism:	TA100		
		No. of Revertants						
Test Item	Treatment	R1	R2	R3	Mean	SD	Observation	Fold increase
Untreated control	**-**	178	197	194	**190**	**10.2**	NR, NP	-

**Without S9**								

Adventol (50% v/v)	0	226	189	178	**198**	**25.1**	NR, NP	-
Synacinn™ (mg/plate)	0.313	181	224	215	**207**	**22.7**	NR, NP	**1.05**
	0.625	153	186	191	**177**	**20.6**	NR, NP	**0.89**
	1.25	163	223	184	**190**	**30.4**	NR, NP	**0.96**
	2.5	185	185	203	**191**	**10.4**	NR, NP	**0.96**
	5	187	188	182	**186**	**3.2**	NR, NP	**0.94**
2AA (µg/plate)	5	179	211	186	**192**	**16.8**	NR, NP	**1.01**
Sodium azide (µg/plate)	5	1650	1580	1768	**1666**	**95.0**	NR, NP	**8.77**

**With S9**								

Adventol (50% v/v)	0	213	225	235	**224**	**11.0**	NR, NP	-
Synacinn™ (mg/plate)	0.313	214	220	211	**215**	**4.6**	NR, NP	**0.96**
	0.625	198	207	186	**197**	**10.5**	NR, NP	**0.88**
	1.25	201	221	208	**210**	**10.1**	NR, NP	**0.94**
	2.5	223	230	209	**221**	**10.7**	NR, NP	**0.99**
	5	207	219	220	**215**	**7.2**	NR, NP	**0.96**
2AA (µg/plate)	5	2636	2260	2060	**2319**	**292.4**	NR, NP	**12.21**

2AA: 2-Aminoanthracene; NR: No reduction in bacterial background lawn; NP: No precipitation

**Table 8:** Mutagenicity assay 1 by pate incorporation method on TA102.

Mutagenicity assay 1 - Plate Incorporation					Organism:	TA102		
		No. of Revertants						
Test Item	Treatment	R1	R2	R3	Mean	SD	Observation	Fold increase
Untreated control	**-**	464	474	481	**473**	**8.5**	NR, NP	-

**Without S9**								

Adventol (50% v/v)	0	561	535	517	**538**	**22.1**	NR, NP	-
Synacinn™ (mg/plate)	0.313	477	474	495	**482**	**11.4**	NR, NP	**0.90**
	0.625	494	550	537	**527**	**29.3**	NR, NP	**0.98**
	1.25	543	551	530	**541**	**10.6**	NR, NP	**1.01**
	2.5	510	539	476	**508**	**31.5**	NR, NP	**0.94**
	5	502	528	550	**527**	**24.0**	NR, NP	**0.98**
Ametycin (µg/plate)	0.5	2110	1980	1896	**1995**	**107.8**	NR, NP	**4.22**

**With S9**								

Adventol (50% v/v)	0	517	498	540	**518**	**21.0**	NR, NP	-
Synacinn™ (mg/plate)	0.313	499	512	530	**514**	**15.6**	NR, NP	**0.99**
	0.625	552	560	440	**517**	**67.1**	NR, NP	**1.00**
	1.25	544	541	582	**556**	**22.9**	NR, NP	**1.07**
	2.5	507	486	520	**504**	**17.2**	NR, NP	**0.97**
	5	465	538	480	**494**	**38.6**	NR, NP	**0.95**
2AA (µg/plate)	10	2012	1996	1890	**1966**	**66.3**	NR, NP	**4.16**

2AA: 2-Aminoanthracene; NR: No reduction in bacterial background lawn; NP: No precipitation

**Table 9:** Mutagenicity assay 2 by pre-incubation method on TA1537.

Mutagenicity assay 2 – Pre-incubation					Organism:	TA1537		
		No. of Revertants						
Test Item	Treatment	R1	R2	R3	Mean	SD	Observation	Fold increase
Untreated control	**-**	4	4	9	**6**	**2.9**	NR, NP	-

**Without S9**								

Adventol (50% v/v)	0	8	7	4	**6**	**2.1**	NR, NP	-
Synacinn™ (mg/plate)	0.313	6	10	3	**6**	**3.5**	NR, NP	**1.00**
	0.625	5	4	6	**5**	**1.0**	NR, NP	**0.83**
	1.25	5	10	4	**6**	**3.2**	NR, NP	**1.00**
	2.5	9	5	3	**6**	**3.1**	NR, NP	**1.00**
	5	9	7	5	**7**	**2.0**	NR, NP	**1.17**
ICR-191 (µg/plate)	1	210	225	197	**211**	**14.0**	NR, NP	**35.17**

**With S9**								

Adventol (50% v/v)	0	14	7	8	**10**	**3.8**	NR, NP	-
Synacinn™ (mg/plate)	0.313	9	14	8	**10**	**3.2**	NR, NP	**1.00**
	0.625	5	11	8	**8**	**3.0**	NR, NP	**0.80**
	1.25	4	10	13	**9**	**4.6**	NR, NP	**0.90**
	2.5	9	10	6	**8**	**2.1**	NR, NP	**0.80**
	5	11	4	7	**7**	**3.5**	NR, NP	**0.70**
2AA (µg/plate)	5	188	214	175	**192**	**19.9**	NR, NP	**32.00**

2AA: 2-Aminoanthracene; NR: No reduction in bacterial background lawn; NP: No precipitation

**Table 10:** Mutagenicity assay 2 by pre-incubation method on TA1535.

Mutagenicity assay 2 – Pre-incubation					Organism:	TA1535		
		No. of Revertants						
Test Item	Treatment	R1	R2	R3	Mean	SD	Observation	Fold increase
Untreated control	**-**	18	18	14	**17**	**2.3**	NR, NP	-

**Without S9**								

Adventol (50% v/v)	0	10	20	20	**17**	**5.8**	NR, NP	-
Synacinn™ (mg/plate)	0.313	16	17	20	**18**	**2.1**	NR, NP	**1.06**
	0.625	13	13	9	**12**	**2.3**	NR, NP	**0.71**
	1.25	12	17	11	**13**	**3.2**	NR, NP	**0.76**
	2.5	13	11	27	**17**	**8.7**	NR, NP	**1.00**
	5	17	13	14	**15**	**2.1**	NR, NP	**0.88**
Sodium azide (µg/plate)	5	1210	1197	1224	**1210**	**13.5**	NR, NP	**71.18**

**With S9**								

Adventol (50% v/v)	0	7	7	9	**8**	**1.2**	NR, NP	-
Synacinn™ (mg/plate)	0.313	5	10	10	**8**	**2.9**	NR, NP	**1.00**
	0.625	11	7	7	**8**	**2.3**	NR, NP	**1.00**
	1.25	6	7	12	**8**	**3.2**	NR, NP	**1.00**
	2.5	13	6	8	**9**	**3.6**	NR, NP	**1.13**
	5	5	12	14	**10**	**4.7**	NR, NP	**1.25**
2AA (µg/plate)	5	183	152	208	**181**	**28.1**	NR, NP	**10.65**

2AA: 2-Aminoanthracene; NR: No reduction in bacterial background lawn; NP: No precipitation

**Table 11:** Mutagenicity assay 2 by pre-incubation method on TA98.

Mutagenicity assay 2 – Pre-incubation					Organism:	TA98		
		No. of Revertants						
Test Item	Treatment	R1	R2	R3	Mean	SD	Observation	Fold increase
Untreated control	**-**	66	53	60	**60**	**6.5**	NR, NP	-

**Without S9**								

Adventol (50% v/v)	0	59	60	71	**63**	**6.7**	NR, NP	-
Synacinn™ (mg/plate)	0.313	47	64	61	**57**	**9.1**	NR, NP	**0.90**
	0.625	40	53	45	**46**	**6.6**	NR, NP	**0.73**
	1.25	42	53	64	**53**	**11.0**	NR, NP	**0.84**
	2.5	38	55	43	**45**	**8.7**	NR, NP	**0.71**
	5	61	90	75	**75**	**14.5**	NR, NP	**1.19**
2-Nitrofluorene (µg/plate)	5	958	846	1024	**943**	**90.0**	NR, NP	**15.72**

**With S9**								

Adventol (50% v/v)	0	47	39	35	**40**	**6.1**	NR, NP	-
Synacinn™ (mg/plate)	0.313	27	44	37	**36**	**8.5**	NR, NP	**0.90**
	0.625	34	33	35	**34**	**1.0**	NR, NP	**0.85**
	1.25	39	32	40	**37**	**4.4**	NR, NP	**0.93**
	2.5	30	39	35	**35**	**4.5**	NR, NP	**0.88**
	5	46	32	45	**41**	**7.8**	NR, NP	**1.03**
2AA (µg/plate)	5	1628	1438	1310	**1459**	**160.0**	NR, NP	**24.32**

2AA: 2-Aminoanthracene; NR: No reduction in bacterial background lawn; NP: No precipitation

**Table 12:** Mutagenicity assay 2 by pre-incubation method on TA100.

Mutagenicity assay 2 – Pre-incubation					Organism:	TA100		
		No. of Revertants						
Test Item	Treatment	R1	R2	R3	Mean	SD	Observation	Fold increase
Untreated control	**-**	134	132	138	**135**	**3.1**	NR, NP	-

**Without S9**								

Adventol (50% v/v)	0	181	172	158	**170**	**11.6**	NR, NP	-
Synacinn™ (mg/plate)	0.313	126	184	188	**166**	**34.7**	NR, NP	**0.98**
	0.625	160	143	142	**148**	**10.1**	NR, NP	**0.87**
	1.25	192	140	140	**157**	**30.0**	NR, NP	**0.92**
	2.5	175	157	153	**162**	**11.7**	NR, NP	**0.95**
	5	180	128	152	**153**	**26.0**	NR, NP	**0.90**
2AA (µg/plate)	5	157	180	174	**170**	**11.9**	NR, NP	**1.26**
Sodium azide (µg/plate)	5	1478	1528	1618	**1541**	**70.9**	NR, NP	**11.41**

**With S9**								

Adventol (50% v/v)	0	192	178	169	**180**	**11.6**	NR, NP	-
Synacinn™ (mg/plate)	0.313	199	187	163	**183**	**18.3**	NR, NP	**1.02**
	0.625	177	197	125	**166**	**37.2**	NR, NP	**0.92**
	1.25	160	161	162	**161**	**1.0**	NR, NP	**0.89**
	2.5	158	171	172	**167**	**7.8**	NR, NP	**0.93**
	5	171	177	153	**167**	**12.5**	NR, NP	**0.93**
2AA (µg/plate)	5	1688	1854	1724	**1755**	**87.3**	NR, NP	**13.00**

2AA: 2-Aminoanthracene; NR: No reduction in bacterial background lawn; NP: No precipitation

**Table 13:** Mutagenicity assay 2 by pre-incubation method on TA102.

Mutagenicity assay 2 – Pre-incubation					Organism:	TA102		
		No. of Revertants						
Test Item	Treatment	R1	R2	R3	Mean	SD	Observation	Fold increase
Untreated control	**-**	469	336	472	**426**	**77.7**	NR, NP	-

**Without S9**								

Adventol (50% v/v)	0	453	432	439	**441**	**10.7**	NR, NP	-
Synacinn™ (mg/plate)	0.313	479	489	451	**473**	**19.7**	NR, NP	**1.07**
	0.625	462	458	432	**451**	**16.3**	NR, NP	**1.02**
	1.25	451	488	477	**472**	**19.0**	NR, NP	**1.07**
	2.5	470	410	452	**444**	**30.8**	NR, NP	**1.01**
	5	459	471	444	**458**	**13.5**	NR, NP	**1.04**
Ametycin (µg/plate)	0.5	1782	1988	2058	**1943**	**143.5**	NR, NP	**4.47**

**With S9**								

Adventol (50% v/v)	0	371	359	427	**386**	**36.3**	NR, NP	-
Synacinn™ (mg/plate)	0.313	432	381	427	**413**	**28.1**	NR, NP	**1.07**
	0.625	312	387	388	**362**	**43.6**	NR, NP	**0.94**
	1.25	400	389	408	**399**	**9.5**	NR, NP	**1.03**
	2.5	401	378	397	**392**	**12.3**	NR, NP	**1.02**
	5	429	398	414	**414**	**15.5**	NR, NP	**1.07**
2AA (µg/plate)	10	1688	1498	1824	**1670**	**163.7**	NR, NP	**3.92**

2AA: 2-Aminoanthracene; NR: No reduction in bacterial background lawn; NP: No precipitation

## 2 Experimental Design, Materials and Methods

### 2.1 Formulation analysis

Synacinn^TM^ powder was provided by Proliv Life Sciences Sdn Bhd and standardized with five selected markers as follows: rosmarinic acid – 0.41% w/w, andrographolide – 0.25% w/w, catechin – 1.48% w/w, curcumin – 0.11% w/w and gallic acid – 1.56% w/w. Andrographolide (sc-205594A), catechin (sc-204673A), curcumin (sc-200509A) and gallic acid (sc-205704A) were purchased from Santa Cruz Biotechnology, while rosmarinic acid (sc-202796A) was purchased from Chengdu Biopurify Phyto Chemicals Ltd. All test items were stored at room temperature protected from light. Identification of Synacinn™ markers was done for the formulation analysis by comparing the retention time of each marker in all the dose strengths against the identification solution. Only one marker (catechin) was quantified by reporting the peak area and Percentage Relative Standard Deviation (%RSD= Standard Deviation/ Mean x 100; acceptance limit of %RSD ≤ 20%) to evaluate homogeneity of the formulations [1]. All formulations (6.25, 12.5, 25, 50 mg/mL) were pipetted out from top, middle and bottom layer in triplicates, injected once into HPLC and chromatograms were recorded.

#### 2.1.1 Marker stock solution preparation

Each marker was weighed (2 mg) transferred into five separate 10 mL volumetric flask. 5 mL of methanol was added to each flask to dissolve completely and made up the volume to 10 mL with water and mix well.

#### 2.1.2 Marker solution preparation (0.01 mg/mL)

Each of the marker stock solution was pipetted out (1.0 mL) and transferred into 20 mL volumetric flask. The volume was made up to 20 mL with diluent and mixed well.

#### 2.1.3 Identification solution preparation

Synacinn was weighed (50 mg) and transferred into 10 mL volumetric flask. 5 mL of diluent was added and each of the marker stock solution was spiked (0.5 mL) into 10 mL volumetric flask. The volume was made up to 10 mL with diluent and mixed well. The solution was centrifuged at 5000 rpm × 3360 *g* for 5 minutes. The supernatant solution was transferred into HPLC vials and injected into HPLC.

#### 2.1.4 High-Performance Liquid Chromatography (HPLC)

HPLC analysis of Synacinn^TM^ and five markers was performed using Waters Alliance™ HPLC system (Waters, USA). Methanol and water in 1:1 (v/v) ratio was used as diluent. Column used was Zodiac C18 (250 × 4.6 mm; Zodiac Life Sciences, India) with diameter of 5µm. The gradient flow for Synacinn^TM^ were (minutes/% mobile phase B); 0/5%, 12/20%, 15/50%, 20/80%, 25/80%, 32/20%, 32.1/5% and 35/5 % (A: Water: 0.5% formic acid in MeOH: 90:10; B: Water: 0.5% formic acid in MeOH: 10:90). The flow rate and column temperature were 1.0 min/mL and 35 °C±5 °C, respectively. All biomarkers were detected at the wavelength of 254 nm, except for catechin, 280 nm, with injection volume of 50 µL. The total run time was 35 minutes.

### 2.2 Test System

*Salmonella typhimurium* strains of TA1537, TA1535, TA98, TA100 and TA102 were used in this experiment as per the test guidelines OECD 471 and ICH S2 (R1) [2], [3]. Each tester strain was characterized and confirmed on their genotypes. The integrity of the tester strains was tested by verifying its histidine requirement, sensitivity to UV radiation, resistance to ampicillin/ tetracycline and rfa mutation. Only qualified batches of strains were employed in the experiments. All tester strains were maintained as frozen permanent stocks (Cryovial working stocks) and stored in ultra-deep freezer at approximately -70 °C. Frozen aliquots of bacterial culture were thawed and a fixed inoculum was added to a flask containing Oxoid Nutrient Broth (ONB-2). Inoculated flask was incubated overnight (15-16 h) in an incubator equipped with a shaker at 37 °C with shaking [110 revolutions per minute (rpm)]. These overnight grown cultures containing 10^9^ CFU/mL were used for the assay conduct. Optical density was determined using spectrophotometer at 650 nm. Actual cell titers were determined by viable count on nutrient agar plates for each assay and recorded as raw data.

#### 2.2.1 Vehicle selection

Preliminary test was conducted to select appropriate vehicle for the experiment and to assess test item solubility. For vehicle selection, small amount of test item was taken to achieve 50 mg/mL concentration. Test item solubility was checked in different vehicles in preferential order starting with water, DMSO and 50% adventol. Test item did not form clear solution in any of the tested solvents. The formulations were vortexed for 5-10 minutes and centrifuged at 1000 rpm for 5 minutes to remove debris or non-active polysaccharides from purified formulation (supernatant) containing selected makers [4]. The supernatant from each formulation was carefully collected and submitted for analytical method feasibility. Formulation prepared in adventol 50% was most suitable for identification of all the five markers. Therefore, based on the analytical method feasibility, adventol 50 % v/v in purified water was preferred over water and DMSO and selected vehicle for this experiment. Moreover, adventol (99% ethanol) is one of the recommended solvents, biocompatible to *Salmonella* tester strains [5], [6]. Adventol (manufactured by Advent Chembio Private Limited) was stored at room temperature.

### 2.3 Metabolic Activation System

#### 2.3.1 Mammalian Liver Post Mitochondrial Fraction (S9)

The mammalian liver post mitochondrial fraction (S9) used for metabolic activation was obtained from Molecular Toxicology Incorporated, USA (MolToxTM S9), where it was prepared from male Sprague Dawley rats induced with Aroclor 1254. Batches of MolToxTM S9 were stored frozen at -70 ± 10 °C and thawed just prior to use. Each batch was checked by the manufacturer for sterility, protein content, ability to convert known promutagens to bacterial mutagens and cytochrome P 450 catalysed enzyme activities (alkoxyresorufin O dealkylase activities) [7], [8], [9].

#### 2.3.2 Preparation of S9 Mix

S9 mix was prepared freshly for each assay by mixing commercially procured S9 fraction with the required cofactors in the ratio of 1:9 which corresponds to 10% v/v of S9 in the final mixture. Cofactor solution were prepared, filtered and aliquoted as per the requirement and stored approximately at -20 °C. Once prepared, S9 mix was maintained on ice throughout its use during the experiment and left over was discarded. The composition of the cofactors used is given in the following Table 14:

**Table 14:** Composition of cofactors.

Name	Quantity/L
D-Glucose-6-phosphate	1.6 g
Nicotinamide adenine dinucleotide phosphate (NADP)	3.5 g
Magnesium chloride (MgCl_2_)	1.8 g
Potassium chloride (KCl)	2.7 g
Sodium phosphate, dibasic (Na_2_HPO_4_)	11. 4 g
Sodium phosphate, monobasic (NaH_2_PO_4_.H_2_O)	2.8 g
Water	q.s to make up 1 L

### 2.4 Positive Controls Information

The details of the positive controls used were given in the following Table 15;

**Table 15:** Positive controls.

				Use	

Chemical name & CAS No.	Source	Concentration (µg/plate)	Solvent	Strain(s)	S9
2-Nitrofluorene (607-57-8)	Sigma Aldrich	5	DMSO	TA98	−
Sodium azide (26628-22-8)	Sigma Aldrich	5	DMSO	TA100 TA1535	−
ICR-191 (17070-45-0)	Sigma Aldrich	1	DMSO	TA1537	−
Ametycin (Mitomycin C) (50-07-7)	Chempure	0.5	Sterile water	TA102	−
2-Aminoanthracene (613-13-8)	Sigma Aldrich	5	DMSO	TA98 TA100 TA1535 TA1537	+
		10		TA102	

### 2.5 Plating procedure

#### 2.5.1 Plate incorporation method

In this method, the plating was achieved by the following sequence of additions to 2 mL of molten agar (supplemented with 10% v/v, 0.5 mM Histidine-Biotin solution) maintained at 45 ± 2 °C for treatment without metabolic activation:

0.05/0.1 mL of Synacinn™ or positive or vehicle control solution
0.5 mL of Phosphate buffer solution
0.1 mL of bacterial culture

These additions were followed by rapid mixing and pouring on to pre-labelled minimal glucose agar plates. When the agar is set, the plates were inverted and incubated. In case of treatment in presence of metabolic activation, 0.5 mL of S9 cofactor mix was used instead of Phosphate buffer. For untreated (i.e. organism) control, 0.1 mL of respective tester strain was added to 2 mL of top agar followed by rapid mixing and pouring on to pre-labelled minimal glucose agar plates. All the treated plates were incubated at 37 ± 1 °C for 48 to 72 hours and evaluated at the end of incubation.

#### 2.5.2 Pre-incubation method

For Pre-incubation method, a pre-incubation step was included in which, test item solution or control solution, S9 mix or phosphate buffer and bacterial culture were mixed and incubated for 20 minutes at 37 ± 1 °C in a shaking water bath at 75 rpm, then 2 mL of molten agar maintained at 45 ± 2 °C was added to this mixture. The plating and incubation procedure was as described in the routine plate incorporation procedure.

### 2.6 Study design

This study was conducted in two phases viz., Phase I-Dose Range Finding experiment (DRF), Phase II-Mutagenicity Assay 1 and 2, with the reference to the guidelines on the Assessment of Genotoxicity of Herbal Substances/Preparations (EMEA/HMPC/107079/2007), OECD 471 and ICH S2 (R1) [2], [3], [10].

#### 2.6.1 Phase I- Dose Range Finding study (DRF)

This phase was designed to assess the cytotoxicity and precipitation with an objective to select test concentrations for the Phase II-Mutagenicity Assay. DRF was conducted with plate incorporation method using TA100 both with and without metabolic activation (10% S9 cofactor mix) at 8 test concentrations ranging from 0.039 to 5 mg/plate separated by factor of 2. Each control (untreated/vehicle/positive) and test item concentrations will be run in triplicate plates.

#### 2.6.2 Phase II- Mutagenicity assay 1 & 2

The objective of this phase was to evaluate the mutagenic potential of the test item.

Mutagenicity assays were carried out using *S. typhimurium* tester strains TA1535, TA1537, TA98, TA100 and TA102. This experiment was conducted both with and without metabolic activation system (10% S9 cofactor mix) using five test concentrations. Each control (untreated/vehicle/positive) and test item concentrations were run in triplicate plates. Mutagenicity assay 1 and 2 were performed using plate incorporation and pre-incubation methods, respectively. Based on the DRF data, concentrations for the Phase II experiments were selected and provided in the following Table 16;

**Table 16:** Synacinn™ – Mutagenicity assay.

Phase II	Concentration of test item solution(mg/mL)	Volume of test item solution per culture (µL)	Final concentration (mg/plate)
Mutagenicity assay 1 (Plate incorporation method)	50 25 12.5 6.25 6.25	100 100 100 100 50	5 2.5 1.25 0.625 0.313
Mutagenicity assay 2 (Pre-incubation method)			

Final concentrations represented up to 3 decimals only. In order to obtain all test doses within the validated bracketed range of 5 to 50 mg/mL for dose formulation analysis, test volume was adjusted to 50 µL of 6.25 mg/mL to achieve 0.313 mg/plate.

## CRediT Author Statement

**Siti Nurazwa Zainol**: Investigation; **Anis Fadhlina**: Writing- Original draft preparation; **Sri Vijaya Rentala:** Formal analysis, Investigation; **Manjula Yalaka:** Formal analysis, Investigation; **Leela Krishna Vatsavai:** Project administration; **Renuka Pillai:** Formal analysis, Investigation; **Hassan Fahmi Ismail:** Review & editing; **Fadzilah Adibah Abdul Majid**: Conceptualization, Supervision.

## Declaration of Competing Interest

The authors declare that the article content was composed in the absence of any commercial or financial relationships that could be construed as a potential conflict of interest. The following authors; Siti Nurazwa Zainol and Fadzilah Adibah Abdul Majid are affiliated to Proliv Life Sciences SDN. BHD. The following authors; Sri Vijaya Rentala, Manjula Yalaka, Leela Krishna Vatsavai and Renuka Pillai are affiliated to Aurigene Pharmaceutical Services Limited. All authors confirm that the results of this experiments are not influenced by the authors' affiliation to the stated companies.

## Appendix Supplementary materials

Image, application 1
